# Effectiveness of a psycho-educational intervention to prevent postpartum parental distress and enhance infant well-being: study protocol of a randomized controlled trial

**DOI:** 10.1186/s13063-017-2348-y

**Published:** 2018-01-04

**Authors:** Marjolein Missler, Roseriet Beijers, Jaap Denissen, Annemieke van Straten

**Affiliations:** 1 Department of Clinical, Neuro and Developmental Psychology and Amsterdam Public Health Research Institute, Vrije Universiteit Amsterdam, Amsterdam, Netherlands; 2 Department of Developmental Psychology, Tilburg University, Tilburg, Netherlands; 3 Behavioural Science Institute, Radboud University, Nijmegen, Netherlands

**Keywords:** Parents, Pregnant women, Stress, Early intervention, Prevention, Infant health

## Abstract

**Background:**

The first months after birth can be challenging for parents, leading to parental distress and decreased well-being. Parents with high levels of distress are found to respond less adequately and sensitively to their infant, which in turn affects infant well-being and health. The goal of this study is to examine the effectiveness of a psycho-educational intervention to prevent postpartum parental distress and enhance the quality of caregiving and infant well-being. In contrast to other interventions, this intervention will be (1) offered already before birth, (2) offered to all parents-to-be, regardless of their risk of postpartum distress, and (3) include fathers. The proposed study examines the effectiveness of this intervention on (1) parenting distress, (2) quality of caregiving, and (3) the infant’s well-being.

**Methods/design:**

In this randomized controlled trial, 128 pregnant women and their partners will be recruited through midwifery practices and general media. Women with a complicated pregnancy, current psychopathology, insufficient Dutch language proficiency and without Internet access will be excluded. Parents will be randomized to either the intervention or a waitlist control group. The intervention consists of a booklet and video (offered prenatally), a home visit at 34–36 weeks of pregnancy and a telephone call 4 weeks after birth. Information and practical tools are provided on (1) sensitive responding and making contact with the baby, (2) crying, (3) feeding, and (4) sleeping. Assessments will take place at baseline (26–34 weeks of pregnancy), during the home visit (34–36 weeks of pregnancy), and 2, 6, and 10 weeks after birth. The control group will be offered the intervention after the end of the study. The primary outcome is maternal parenting stress. Secondary outcomes are: paternal parenting stress, parental well-being, quality of caregiving, and infant well-being and health.

**Discussion:**

The goal of this study is to test the effects of a psycho-educational prenatal parenting intervention to prevent postpartum parental distress and to enhance well-being in both parents and infants. When the intervention appears effective it can be implemented broadly because of its low costs. It will make support available for a large number of parents and their children.

**Trial registration:**

Netherlands National Trial Register, ID: NTR6065. Registered on 15 September 2016.

**Electronic supplementary material:**

The online version of this article (10.1186/s13063-017-2348-y) contains supplementary material, which is available to authorized users.

## Background

Having a baby is an intense experience that turns the world of new parents upside down. While this life event often brings joy and happiness, the first months can also be challenging for parents (van Scheppingen, Denissen, Chung, Tambs, and Bleidorn, in press [61]). Parents have to develop a range of new skills in taking care for their infant, which requires extra effort and energy, while at the same time they have to deal with a significant lack of sleep. It is not surprising, therefore, that roughly 14% of mothers and 10% of fathers experience moderate or severe levels of postpartum distress [43], mainly consisting of depressive symptoms (e.g., [41]). This study examines the effectiveness of a psycho-educational intervention to prevent postpartum parental distress and enhance infant well-being.

### Parental distress and sensitive responding to the infant’s needs

Parental distress (including, but not limited to, stress related to the parental role) has been related to decreased quality of caregiving. Both maternal [11, 24] as well as paternal depression [46] have been associated with a range of negative outcomes for the child’s emotional, cognitive and behavioral development [21]. The supposed mechanism underlying this link is that depressed parents are less able to respond sensitively to their infant [24, 37]. Sensitivity refers to the extent to which caregivers timeously and appropriately respond to the infant’s needs and signals [2]. Caregiver sensitivity has been identified as one of the most robust predictors of the development of a secure attachment bond between parent and child [3, 20, 30, 55]. Moreover, sensitivity has also been associated with increased social competence, resilience, regulatory capacities, and lower stress levels later in life [23, 25, 50, 52].

Other important characteristics of caregiving behavior are the type of feeding and the infant sleeping location that parents have arranged. Current Dutch guidelines advise parents to exclusively breastfeed for 6 months, and to share their room with the infant for 6 months (infant sleeping in its own crib in the parents’ room, but not in the parental bed) [56, 57]. The World Health Organization (WHO) also recommends exclusive breastfeeding until the infant is 6 months of age [63]. Breastfeeding has been related to important health benefits for the child, including protection against of infections, overweight, and diabetes [28, 62]. Moreover, breastfeeding has been related to increased cognitive development [29]. Next to breastfeeding, there is international agreement on the benefits of room-sharing during the first 6 months of the infant’s life. Room-sharing protects from sudden infant death syndrome (SIDS; [47]). Also, being close to the infant facilitates the process of breastfeeding at night [4, 5]. There are indications that the proximity of the parent functions as a buffer against the infant’s distress [8, 58]. Despite these recommendations, it has been demonstrated that both breastfeeding and room-sharing are frequently discontinued in the first 2 months of the infant’s life [56, 57].

### Interventions to reduce parental distress

There are a number of interventions available that have been proven effective in reducing parental distress ([3, 26, 33, 35, 60]). These interventions mostly start during the postpartum period and are tailored at families from high-risk populations, such as families with low socioeconomic status (SES), parents of an infant born prematurely, foster parents, and parents from children at risk for developing autism spectrum disorder. Moreover, these previous interventions mostly focused on only the mother. This is remarkable because research has shown that the father can play an important role; for example, in supporting the mother with breastfeeding and prolonging the breastfeeding period [38, 44]. Also, intervention studies aimed at treating maternal depression showed that inclusion of the father in treatment led to greater successes of the treatment [54]. Paternal support might protect against maternal stress and the development of postpartum depression, and mitigate the negative effects of depression on the mother-infant interaction [60]. Furthermore, it has also been found that fathers tend to decrease their involvement in childcare when they suffer from psychological adjustment difficulties around the transition to parenthood [31]. Thus, previous research has pointed to the importance of including fathers in the intervention as this might have positive effects on the well-being of both parents as well as on the quality of their caregiving.

To our knowledge, there is only one intervention to date that focused on parents from both low- and high-risk groups. This intervention consists of psycho-education about infant sleep and crying patterns, in combination with a telephone consult and a parent group, and showed positive results in a randomized controlled trial [27]. However, the intervention did not include information on sensitivity or feeding, and it was offered postpartum. Furthermore, as only data of the primary caregiver was collected, almost only data from the mother was available as a result.

### The current study

We developed an intervention which can be offered during pregnancy, to both mothers and fathers, regardless of their risk of postpartum distress. The intervention consists of psycho-education (booklet and video) as well as a prenatal home visit and a postpartum telephone call.

**Fig. 1:**
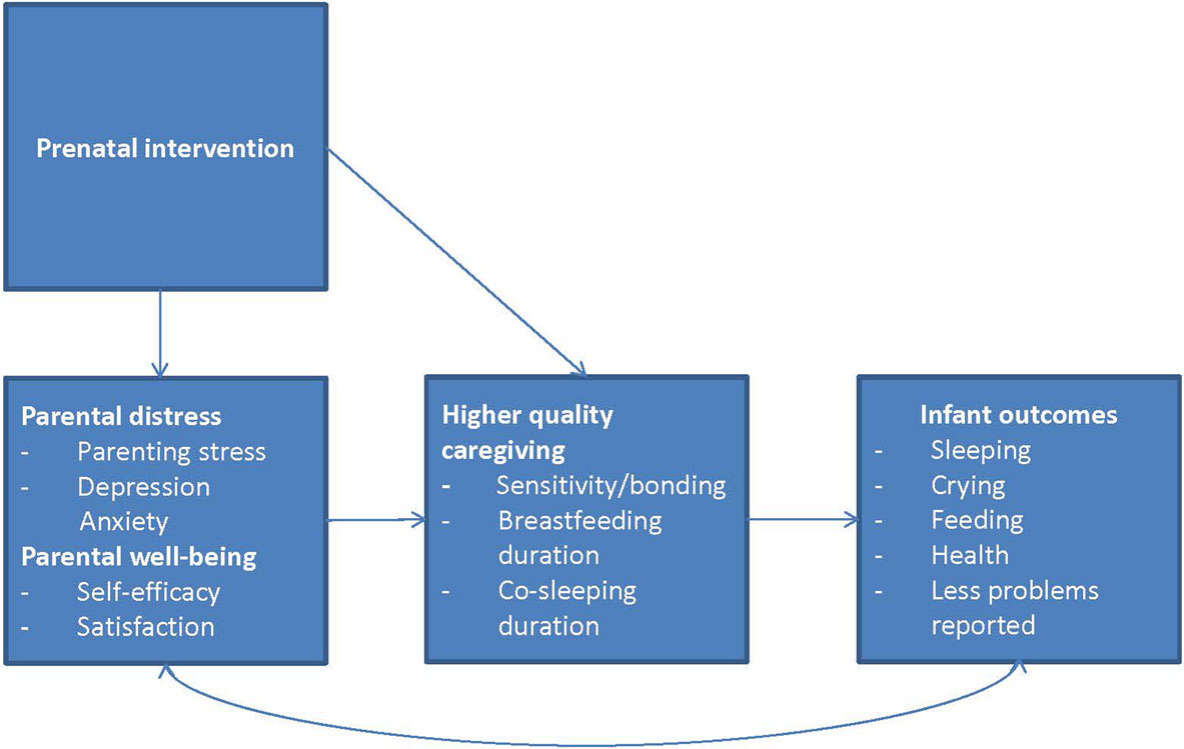
Primary and secondary study outcomes (jpeg)

We expect that the proposed intervention will reduce maternal parenting stress. Furthermore, we expect that the intervention will reduce paternal parenting stress and parental distress in general. Moreover, we expect that parental well-being will be enhanced. By psycho-educating parents during pregnancy, we expect parents to experience more self-efficacy and satisfaction in fulfilling their roles. In this way, parents should be better able to provide high-quality caregiving (including breastfeeding and co-sleeping), leading to enhanced infant well-being (less problems with sleeping, crying and feeding, and better well-being and health) (Fig. 1).

### Objectives

The primary objective of this study is to determine the postpartum effect of the intervention on maternal parenting stress. Secondary outcomes are: paternal parenting stress, parental distress in general (anxiety and depression) and parental well-being (satisfaction with the parental role, and self-efficacy in caring for the infant); quality of caregiving (bonding, breastfeeding, and co-sleeping); and infant well-being and health (crying, feeding, sleeping, well-being, and health).

## Methods/design

### Trial design

**Fig. 2:**
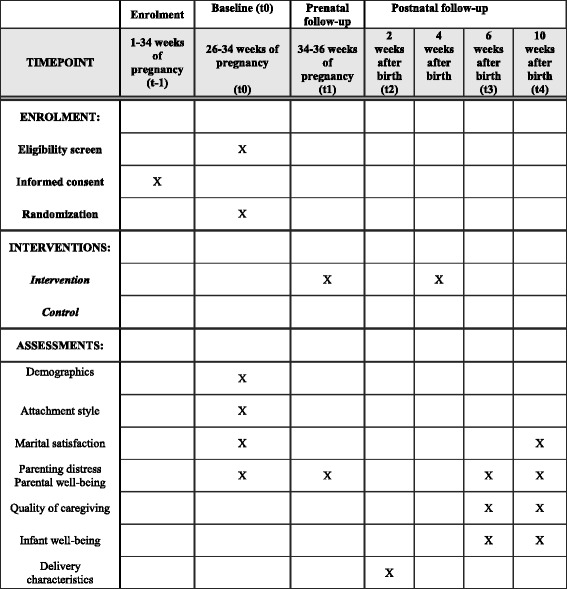
Standard Protocol Items: Recommendations for Interventional Trials (SPIRIT) Figure with trial design and outcome assessments. After screening for eligibility, participants are asked to sign an informed consent form and to complete the baseline questionnaire. Then, participants are randomized in either the intervention or the control group. The intervention starts with a home visit (at t1) and a postnatal telephone call (4 weeks after birth). Measurements take place at 26–34 weeks of pregnancy (baseline; t0); 34–36 weeks of pregnancy (t1); 2 weeks after birth (t2; delivery characteristics); 6 weeks after birth (t3); and 10 weeks after birth (t4)

The study is a randomized controlled trial with two parallel groups: an intervention and a waitlist control group. The waitlist group will receive the intervention after the last assessment (10 weeks after the birth of their infant, see Fig. 2). Please refer to Additional file 1 for the Standard Protocol Items: Recommendations for Interventional Trials (SPIRIT) checklist.

**Fig. 3:**
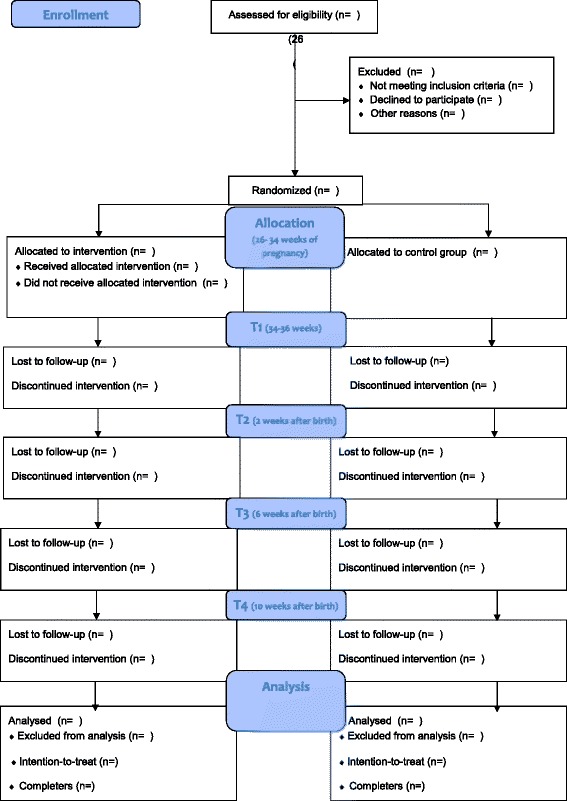
Consolidated Standards of Reporting Trials (CONSORT) flow diagram. After screening for eligibility, participants are randomized in either the intervention or the control group. Follow-up measurements take place at 34–36 weeks of pregnancy (t1); 2 weeks after birth (t2; delivery characteristics); 6 weeks after birth (t3); and 10 weeks after birth (t4)

### Study setting and recruitment

Women will be recruited either through midwifery practices or general media (newspaper ads and Internet banners). The midwives will give the parents-to-be a flyer, referring to the study’s website. Alternatively, parents can contact us directly by responding to online advertisements in which a link to the website is provided. On the website, a registration form can be filled out. Upon registering, parents will receive the digital information brochure of the study and an informed consent form. Following the Declaration of Helsinki, it will be clearly indicated on the informed consent form that study participation is entirely voluntary and that participants can withdraw from participation at any time without any negative consequences for them or their child. If parents decide to take part in the study, they will be asked to return the informed consent form. Interested parents who are not eligible for inclusion will receive a message explaining the reason(s) for their exclusion.

### Inclusion and exclusion criteria

Pregnant women will be included in the study when they are before the 34th week of pregnancy and do not have severe medical conditions due to pregnancy (i.e., gestational diabetes or pre-eclampsia). Furthermore, women need sufficient Dutch language proficiency to understand the information offered to them during the intervention (through a booklet and a video). Also, access to the Internet is required to be able to complete the online questionnaires. Women with current psychopathology (defined as current treatment for psychopathology or treatment in the 6 months before inclusion) will be excluded. The partners of the pregnant women will also be asked to participate. However, women without a partner, or women with a partner who does not want to participate, can also enter the study.

### Sample size calculation

We expect an effect size of 0.50 (Cohen’s *d*). This is based on the work of Hiscock et al. [27]. Their intervention, aimed at preventing infant sleep and crying problems, showed an odds ratio of 0.57 (converted into a Cohen’s *d* of 0.48) on symptoms of maternal depression in favor of the intervention group. Using a power of 80%, an alpha of 0.05, we need to include 64 participants in both conditions, 128 in total, to answer our primary research question.

### Randomization

An independent researcher will generate random number sequences using random allocation software with a 1:1 ratio. We will use random sequence blocks (blocks between 6 and 8), stratified by birth order (first child/ no first child) and paternal participation (yes/no). An independent researcher will allocate each consenting participant to either the control or the intervention group. Given the nature and design of the study, blinding after randomization is not possible. Participants will receive the outcome of randomization by e-mail. Please refer to Fig. 3 for an overview of participant's flow through the study.

### Intervention

Parents will receive access to the psycho-education materials (booklet and video) directly after randomisation, which is between the 26th and the 34th weeks of pregnancy (depending on when the parents apply for participation). The intervention further consists of two support sessions: one home visit between 34 and 36 weeks of pregnancy and one telephone call 4 weeks after birth.

#### Psycho-educational booklet and video

In general, the intervention conveys the importance of responding sensitively to the needs of the infant. The psycho-educational booklet developed by Hiscock et al. [27] was used as a starting point. We developed and extended the intervention further based on recent empirical research and together with a number of different experts on attachment (researchers), breastfeeding (lactation consultants), and pre and postnatal care for both mother and child (midwives). Our booklet consists of four chapters about: (1) sensitive responding and making contact with the baby, (2) crying, (3) feeding, and (4) sleeping. The first chapter includes information about contact-seeking behavior of the baby and how to interpret infant’s signals of distress and respond to these adequately (e.g., [9]). The chapter also includes information explaining the needs of the infant (such as the need for closeness, skin-to-skin contact, and sensitive care), followed by a discussion about the parent’s own needs and how to attend to these. Then, the booklet continues with a chapter about crying. The normal crying curve is addressed, and different tools are provided on how to sooth a baby. Also, potential causes of excessive crying are discussed. The third chapter about feeding includes information about hunger signals that the baby might convey. Information is given on breastfeeding, and its positive effects for both mother and child. Practical information about the use of formula milk is also provided. The fourth and final chapter on sleeping provides information on average sleeping patterns and sleep signals that a tired baby might convey. Moreover, the importance of room-sharing during the first 6 months will be discussed and suggestions on when and how to support the infant in learning to sleep alone in a separate room are given. It is also important to note here that sensitive care is put central in our intervention, and that, for example, “crying it out” and “camping it out” techniques [32], in which parents are instructed to let infants cry for a specified amount of time, are not included in our intervention. The booklets ends with a section about common baby myths, such as “Keeping the baby awake all day, will make the baby sleep better during the night” or “When I pick up my baby every time, I will spoil the baby.” The booklet consists of written text and pictures. Most importantly, the booklet provides many examples on how to use the information in daily life. In the booklet, it is explained that the provided information is not meant to be prescriptive; rather, it encourages parents to distil those elements that are most helpful for them in their specific situation (while keeping the principles of sensitive care in their mind). This way, parents can start discovering which tools fit best with their and their infant’s preferences and needs.

At the same time as the booklet, participants receive access to an online video. This video is developed by psychologists who are experts in promoting the mental health of babies and their parents by translating academic knowledge into easily accessible interventions (Stichting Babywerk, The Netherlands). The video serves as an illustration of the topics which are described in the booklet and shows practical examples. It is a story format in which the experiences of (upcoming) parents are shown. An expert on infant development comments on each of the fragments. Also, after each fragment, participants are confronted with a thought-provoking question about what they just have seen. The aim of these questions is to stimulate participants to think about the information in relation to their lives. For example, in the story, the upcoming mother is worried that she will be unable to put her mobile phone away when her baby is awake. At this point, the participant will be asked what their thoughts are about this issue. Next, the expert explains why being able to recognize and respond to the infant’s signals is important. After the explanation, the video shows how to notice and respond to an infant seeking eye contact. Watching the video and responding to the questions takes about 15–20 min. The parents are encouraged to put this advice into practice after the birth of their child.

#### Support sessions

Parents will be visited at home during weeks 34–36 of the pregnancy. The primary aim of this home visit is to discuss the provided information (booklet and video) and to answer any questions about the materials the parents might have. The secondary aim is to explain that the provided information is not meant to be prescriptive; rather, we aim to provide parents with a set of tools (the practical examples) that they can choose from. By means of provided materials, we ask participants to start discovering which tools fit best with their and their infant’s preferences and needs.

Four weeks after the birth of their child, parents will be contacted by telephone. During this call, we will ask the parents how they and their child are doing, and whether they are experiencing any problems in implementing the information from the booklet and the video in their daily lives. Also, parents are given the opportunity to ask questions with regard to their infant’s feeding, crying, sleeping, and contact-seeking behavior. They can also ask questions with regard to their own well-being (i.e., experienced stress or feelings of anxiety). Both the home visit as well as the telephone call will be performed by the corresponding author, who has a background in clinical psychology and infant development.

### Waitlist control group and care-as-usual

Parents in the waitlist control group will receive the same materials as the parents in the intervention group after the last assessment (10 weeks after birth). The information can still be of use for them at that time. Both groups, the intervention and control group, will have access to care-as-usual during the postpartum period. In the Netherlands, this consists of regular consults with a specialized nurse at home (2 weeks after birth) or at the well-baby clinic (at 4, 8, and 12 weeks after birth) during which the health of the infant is checked upon and the infant’s growth is followed.

### Assessments and outcomes

We will measure at baseline (26–34 weeks of pregnancy, t0); 34–36 weeks of pregnancy (t1); 2 weeks after birth (t2); 6 weeks after birth (t3); and 10 weeks after birth (t4). All assessments will take place online, except for the completion of the infant behavior diary (t3; Fig. 2).

#### Baseline variables

At baseline (t0), we assess the demographic characteristics of the parents (date of birth; marital status, income, educational level, and working hours).

Furthermore, to control for possible insecure attachment styles of the parents (which can have an impact on their caregiving quality (e.g., [22]), we will measure attachment styles of the parents using the short form of the Experiences in Close Relationships Questionnaire (ECR-short form; [15, 34]^1^). The 12 items of this instrument are derived from the avoidance and anxious attachment subscales of the ECR-R (six items of each subscale; [10]). Response options vary from 1 (strongly disagree) to 7 (strongly agree). The avoidance subscale measures the need to stay independent from others and to avoid intimacy ([34], see also [7]). The anxiety subscale is concerned with the degree to which the subject worries about rejection and abandonment ([7, 34]). Following recoding of items 15, 25, 27, 29, and 31; for each subscale (anxiety and avoidance) an average score of between 1 and 7 can be computed. Higher scores reflect more attachment anxiety and avoidance. The ECR-short form showed good psychometric properties in different samples: Lafontaine et al. [34] reported Cronbach’s alpha’s of .78 to .87 for the anxiety subscale and .74 to .83 for the avoidance subscale.

#### Primary outcome

The primary outcome is maternal parenting stress (assessed at t0; t1; t3; and t4) and is measured with 10 items of the Dutch version of the Parenting Stress Index (PSI; [1, 18]). The complete version of this instrument consists of 123 items. A shortened version of 25 items is available [19]. However, since the PSI has been originally developed for parents of children up to 14 years [18], our aim was to select the 10 items that are most relevant for parents of a newborn child. Following the procedure of Missler et al., [39], we selected those items that, in our view, best captured their experience. One item was added because it was considered to be central to the parenting stress construct (at least for parents with very young children): “The responsibility I have for my children weighs on me” Missler et al., [39]. An example item is: “I feel restricted by my responsibilities as a parent.” For the administration before birth (t0 and t1), the items were slightly rephrased to capture possible stress-raising expectations of parenthood: “I expect to feel restricted by my responsibilities as a parent.” Response options vary from 1 (totally disagree) to 6 (totally agree). A total score can be derived by summing the individual item scores. This total score (including the added item) ranges from 11 (no stress) to 66 (very high stress).

#### Secondary outcomes

Secondary outcomes are paternal parenting stress, parental well-being, quality of caregiving, and infant well-being and health.

##### Paternal parenting stress

Paternal parenting stress will be measured with the same 10 items of the Dutch version of the Parenting Stress Index (PSI; [1, 18]) used for measuring maternal parenting stress. For more details, see above.

##### Parental well-being

Parental well-being is defined as: (1) depressive symptoms, (2) symptoms of anxiety, (3) satisfaction with the parental role, (4) parental self-efficacy, and (5) sleep quality and quantity.

1. Depressive symptoms will be measured with the Edinburgh Postnatal Depression Scale (EPDS; [17]; Dutch translation Pop, Komproe, and Van Son, 1992). This scale consists of 10 items. Participants can indicate the experienced frequency of each depression-related statement on a 4-point scale. Items are scored 0, 1, 2, or 3 (items 3, 5, 6, 7,8, 9, and 10 are reverse scored). Total scores range from 0 (no depressed feelings) to 30 (severely depressed feelings). The scale shows good psychometric properties: Pop et al. [45] reported an internal consistency of .82 (Cronbach’s alpha) and sufficient concurrent validity
2. Symptoms of anxiety will be measured with the seven items of the anxiety subscale of the Hospital Anxiety and Depression Scale (HADS; [42, 51]). For each item, participants can indicate on a 4-point scale how much anxiety they experience. Total scores on the anxiety subscale vary from 0 (no anxiety) to 21 (severe anxiety). Spinhoven et al. [51] reported good psychometric properties for the Dutch version
3. Satisfaction with the parental role will be measured with three items of the Dutch translation of the Parenting Stress Index (PSI; [18, 19, 36]). Following the procedure of Missler et al., [39], four items were added to this scale. Adding items was necessary because to our knowledge, no measure of parenting satisfaction currently exists. We thus decided to use this composed scale. An example item is: “I enjoy spending time with my child.” For the administration before birth (t0 and t1), the items were rephrased such that the expectation of parents of their satisfaction with their new role after the birth of their child became central: “I expect I will enjoy spending time with my child”
4. Parents will be asked to rate their efficacy as a parent on a 5-point scale, ranging from 1 (not very good) to 5 (a very good parent; [16, 27, 49])
5. Sleep quality and quantity is measured with two items of the Pittsburgh Sleep Quality Index (PSQI; [12]) adapted by [16]. The quality item is: “Over the last 2 weeks, how would you rate your own sleep quality?” Response options are: “Not nearly good enough”; “Not quite good enough”; “Good enough”; and “More than good enough.” The quantity item is: “Over the last 2 weeks, how would you rate your own sleep quantity?”, with the following response options: “Not nearly enough”; “Not quite enough”; “Enough”; and “More than enough.”

##### Quality of caregiving

Quality of caregiving will be measured by assessing the bonding between parent and child, the duration of breastfeeding, and the duration of room-sharing at 10 weeks postpartum.

1. Bonding will be measured with the Maternal Postnatal Attachment Scale (MPAS; [14, 59]). This scale consists of 19 statements referring to parent-child relationship. Each statement can be answered on a 2-point; 4-point; or 5-point scale. An example item is:“When I am with the baby, I feel tense and fearful.” Total scores range from 19 to 95 and can be computed by summing up individual item scores. Low total scores indicate bonding problems between parent and child. Van Bussel et al. [59] reported a Cronbach’s alpha of .75 of the MPAS when administered 8–12 weeks after birth
2. Breastfeeding will be assessed by measuring the duration (in weeks) of breastfeeding and/or breastfeeding mixed with bottle-feeding
3. Room-sharing will be measured as the duration (in weeks) of room-sharing (the infant sleeps in the room of the parents at night)

#### Infant well-being and health

The well-being of the infant will be measured through a diary (72 h at 6 weeks postpartum) assessing crying, sleeping, and feeding [6]. Parents are supposed to rate precision in 5-min slots using different symbols. The following behaviors will be assessed: asleep, awake, and being content, awake and being fussy, awake and crying, awake and feeding; awake and sucking (thumb/dummy). Parents can also indicate the time and duration of feeding, and the type of feeding (breastmilk (through breastfeeding or bottle) or formula milk). Parents are asked whether the rated 72 h period can be viewed as a “typical” period or not. Furthermore, we will ask parents at 6 and 10 weeks after birth whether they are experiencing a problem with infant sleep (day or night), crying or feeding, and if so, to rate the severity of this problem on a 7-point Likert scale ranging from 1 (hardly any problem) to 7 (a severe problem) [27]. Infant health is measured in terms of somatic indices and somatic problems (at 6 and 10 weeks after birth). Parents are asked about their infant’s weight, length, and head circumference and will also be asked to indicate whether their child has experienced fever, a runny nose, any coughing, ocular inflammation, or diaper rash. Additionally, parents are asked if their child had experienced some other medical condition and whether their child has been using any medication.

#### Birth

Variables related to the delivery and the birth are assessed 2 weeks after birth: birth weight, Apgar score, spontaneous delivery, caesarean section, birth complications. We will test whether there are differences between the intervention and control groups for these variables (measuring possible stress factors during the delivery) in the analyses.

#### Marital satisfaction

Marital satisfaction is measured with the global satisfaction items of The Investment Model Scale (IMS; [48]; Dutch translation: Montgomery, Peeters, Schaufeli, and Panagopoulou, [40]). This scale consists of five items, with answering options varying from 1 (totally disagree) to 9 (totally agree). An example item is: “My relationships fulfills my needs for intimacy.” The total score ranges from 5 (not satisfied) to 45 (very satisfied). Montgomery et al., 1998, report a Cronbach’s alpha of .93. Again, we will test for potential differences on this variable between the two groups.

#### Intervention uptake

Finally, we measure the uptake of the intervention at 10 weeks after birth by asking parents whether they have read and watched the materials before the birth of their child. We also ask them whether they looked into the materials again after the birth of their child. Additionally, we monitor online the duration and frequency of watching (parts) of the video. Furthermore, we ask them to rate the frequency of using the information in their daily lives, with the item: “How many times did you use the information from the booklet, video, or the home visit during the daily care for your baby?” Response options were: “Daily”; “Several times a week”; “About once a week”; “About once every 2 weeks”; “About once a month”; “Never.” We also ask them to rate the usefulness of the booklet, video, and the home visit on a 5-point scale ranging from “Not very useful” to “Very useful.”

### Data management

We will keep one file in which research identifiers are linked to participant’s names and (e-mail) addresses. This file will be encrypted and password protected and only be available to the main researchers. All on- and offline data will be safely stored at the university using research identifier only.

### Statistical analysis

The study will be conducted in adherence to the Consolidated Standards of Reporting Trials (CONSORT) Statement. We will perform intention-to-treat analysis, in which all patients who are randomized will be analyzed (independent of treatment or study completion), as well as completers-only analyses. Completers are defined as participants who completed all measurements. Baseline data will be explored to see if there are differences between the group that remains in the study and the group that drops out.

#### Primary outcome

We will compare the parenting stress scores between the two groups using a mixed multilevel model. This way, we can account for the nested structure of the data (mothers and fathers that belong to the same couple). Including both mothers and fathers in one analysis, will positively affect the power of the analyses. Furthermore, the analysis is also robust for missing data (Tabachnik and Fidell, [53]) and unequal sample sizes (more participating mothers than fathers). We will test interaction terms between parental sex (mother versus father) and intervention group (intervention versus control), to test whether the (possible) effect of the intervention is the same for both mothers and fathers. We will also calculate the between-group effect sizes at 6 (t3) and 10 weeks postpartum (t4). We will calculate Cohen’s *d* by subtracting the two mean scores and dividing them by the pooled standard deviations. A Cohen’s *d* of 0.2 can be assumed small, 0.5 to be moderate, and 0.8 to be large [13].

#### Secondary outcomes

We will repeat the mixed multilevel models, and the calculation of Cohens’ *d*, for all outcomes of parental well-being. Next, to determine the effect of the intervention on the quality of caregiving after birth, we will add higher-quality caregiving as a secondary outcome variable to the model. Furthermore, to test whether prenatal well-being of the parents influences postpartum caregiving quality, we will use parental well-being (measured at 26 and 34 weeks of pregnancy) as a predictor of higher-quality caregiving. Finally, we will determine the effect of prenatal parental well-being on infant outcomes postpartum (mediated by higher-quality caregiving). For each of the outcomes we will calculate the between-group effect size at t3 and t4 and their 95% confidence interval. Gender will be added as an interaction term to the model, to see if there are differences between mothers and fathers.

Demographic data, attachment style of the parents and data related to the delivery and birth (birth weight, Apgar score, spontaneous delivery, caesarean section, birth complications) will be used as control variables in the model.

### Data monitoring

Since the current intervention is a non-pharmacological intervention, it is unlikely that adverse effects due to the intervention will occur. Therefore, installing a Data Monitoring Safety Board does not seem warranted.

### Harms

We consider this study to have negligible risks. One possibility is that the parents become aware of possible postpartum stressors and, therefore, become more stressed instead of less stressed. To prevent this rise of stress levels in both groups, we will take specific action. We will underline to the parents from the intervention group that we do not expect them to implement the intervention perfectly. We will explicitly give them the opportunity to make their own choices regarding the advice given in the booklet. Parents are completely free to determine which of the tools provided in the intervention they would like to use.

For the control group, we will stress that the intervention is a way of extra support, not the solution to prevent problems. They will be granted access 10 weeks after birth.

### Amendments

All substantial amendments will be notified to the Medical Ethics Committee (METC) and to the competent authority.

Non-substantial amendments will not be notified to the accredited METC and the competent authority, but will be recorded and filed by the sponsor.

### Dissemination policy

MM and RB will process the data, and both positive and negative findings will be disclosed, unreservedly. Results will be submitted for publication to peer-reviewed scientific journals. The participating parents as well as the participating midwifery centers will receive updates on the study’s progress. At the end of the project, results and conclusions will be presented to all those involved.

## Discussion

This goal of this study is to examine the effectiveness of a psycho-educational intervention to prevent postpartum parental distress and enhance infant well-being. In contrast to other interventions, this intervention will be offered (1) already before birth, (2) to all parents-to-be, regardless of their risk of postpartum distress, and (3) to include fathers.

We expect parents to experience less distress and higher well-being after the birth of their child compared to parents who do not receive this intervention. In turn, we expect that this will have positive effects on the quality of their caregiving, and ultimately on the infant’s well-being and health, resulting in less parent-reported problems with sleeping, crying, and feeding, and increased infant health.

The intervention is focused on sensitive caregiving; that is, responding timely and adequately to the infant’s needs. The booklet consists of four chapters; on making contact and sensitive responding, crying, feeding, and sleeping. A video is available to illustrate the topics mentioned in the booklet. During a prenatal home visit, the provided materials will be discussed. After birth, parents receive a supportive telephone call. Throughout the intervention, it will be underlined that no part of the intervention is meant to be prescriptive; rather, the intervention has been developed to support parents in choosing those elements that are most helpful for them in their specific situation. Indeed, by offering the intervention we aim to stimulate parents to think about and discuss the information provided, whether or not they will implement everything that they have learned. This way, we expect both parents to be better equipped for the transition to parenthood.

In our view the timing of implementation of our psycho-educational intervention is crucial. By intervening already during pregnancy (and continuing to offer support during the first months after birth), our aim is to prevent or mitigate distress among parents and infants – and related problems with breastfeeding and co-sleeping – during the first hours, days, and weeks of the infant’s life. In this way, the infant’s health and early development can optimally benefit. The intervention is low-cost, easy to implement, and can be distributed on a large scale: a large number of parents and infants can thus be supported during a crucial period of their lives.

### Trial status

Recruitment started in November 2016 and is ongoing. In November 2016, the first participants enrolled. Currently, 59 participants have signed the informed consent form and are thus participating in the study (nine of them have completed the study protocol).

## Additional file

Additional file 1: Standard Protocol Items: Recommendations for Interventional Trials (SPIRIT) checklist. (DOC 122 kb)

## Abbreviations

ECR: Experiences in Close Relationships Questionnaire
EPDS: Edinburgh Postnatal Depression Scale
HADS: Hospital Anxiety and Depression Scale
IMS: The Investment Model Scale
MPAS: Maternal Postnatal Attachment Scale
PSI: Parenting Stress Index
PSQI: Pittsburgh Sleep Quality Index
SES: Socioeconomic status
SIDS: Sudden infant death syndrome
WHO: World Health Organization

## Footnotes

1 Items of the ECR-short form are extracted from the ECR-R [10], and this version has been validated in Dutch [15].
